# Development and Validation of a Method for Determination of 43 Antimicrobial Drugs in Western-Style Pork Products by UPLC-MS/MS with the Aid of Experimental Design

**DOI:** 10.3390/molecules27238283

**Published:** 2022-11-28

**Authors:** Xiaoxuan Yu, Xingqiang Wu, Yujie Xie, Kaixuan Tong, Minglin Wang, Jianhui Li, Chunlin Fan, Hui Chen

**Affiliations:** 1 Chinese Academy of Inspection & Quarantine, No. 11, Ronghua South Road, Beijing 100176, China; 2 College of Food Science and Engineering, Shandong Agricultural University, Taian 271018, China; 3 Waters Technology (Shanghai) Co., Ltd., Beijing 101102, China

**Keywords:** bacon and ham, antimicrobial, QuEChERS, Box–Behnken design, UPLC-MS/MS

## Abstract

Western-style pork products have attracted many modern urban consumers, and these products have rapidly entered the Chinese market. The current hazard analysis of processed meat products mainly focuses on processing hazards (PAHs, microorganisms, and food additives), with less attention to veterinary drug residues. According to the survey results, the residues of antimicrobial drugs (sulfonamides and quinolones) in pork and its products in China are a severe problem, which may cause metabolic reactions, toxic effects, or enhance drug resistance. This study applied a modified QuEChERS method combined with ultra-performance liquid chromatography-tandem mass spectrometry (UPLC-MSMS) to develop a rapid and sensitive method for determining antimicrobial drugs in bacon and ham was successfully evaluated methodologically by EU 2002/657/EC. This study used a three-level, three-factor Box–Behnken design (BBD) to optimize the QuEChERS method by response surface methodology. The excellent linearity of the calibration curve was shown in the corresponding concentration range with a coefficient of determination greater than 0.99. The values of decision limit (CCα) and detection capability (CCβ) were in the range of 10.9–31.3 μg/kg and 11.8–52.5 μg/kg, respectively. The method successfully detected two trace levels of antimicrobial drugs in commercially available samples, including sulfadiazine and moxifloxacin.

## 1. Introduction

In recent years, with the continuous improvement of living standards and the change of consumption structure, China, as the world’s largest pork consumer, has increased the variety of pork processed products, the proportion of consumption has increased significantly, and consumption has almost doubled [1]. Among them, Western-style pork products have attracted many modern urban consumers with their advantages of tender meat, nutrition, and convenience. Their products have rapidly entered the Chinese market and gradually integrated into Chinese catering [2]. The Western-style pork products usually take raw pork as ingredients, and through a series of processes such as salting, smoking, fermentation, and drying treatment, a series of products such as bacon, ham, and sausage with a salty or strong smoky flavor are formed. With the popularization of healthy living, more and more consumers are pursuing pollution-free, residue-free, safe, and nutritious food [3]. Currently, research on the hazard analysis of processed meat products mainly focuses on the harmful substances formed during production and processing, such as heterocyclic amines, polycyclic aromatic hydrocarbons, microorganisms, and food additives [4]. However, there was less concern about the possible presence of veterinary drug residues in processed meat products.

Veterinary drugs are used in nearly 80% of animals through feed admixture, oral administration, or injection for their cost-effective economic and commercial value. Global consumption of veterinary drugs is estimated to reach 200,235 tons by 2030 [5]. A large part of them is antibacterial drugs (quinolones and sulfonamides) used in the breeding process to promote the rapid growth of animals, improve feed utilization, prevent and control the bacterial diseases of animals or treat mixed infections. However, to pursue economic interests during the process of pig breeding, some breeders give large amounts, high levels, or not according to the rest period will inevitably lead to residues of veterinary drugs in animal-derived food, which has become one of the factors plaguing the food safety of livestock products [6]. From the information available, analyzing the residues of veterinary drugs in pork and its products marketed in China in the past three years, the residues of quinolones and sulfonamides were a severe problem [7]. Researchers have demonstrated that quinolones and sulfonamides residues may cause diseases directly, including metabolic reactions, toxic effects, blood deterioration, carcinogenesis, and teratogenesis [8]. Meanwhile, the most severe problem is the development of antibiotic resistance [9]. To protect both the quality of food and consumer health, some countries and regions: including the United States [10], China [11], and European Union [12], have set strict maximum residue limits (MRLs) for quinolones and sulfonamides in livestock to combat the illegal use of veterinary drugs in farming.

Developing an efficient, accurate, and sensitive analysis method is required to respond to the level of MRLs established by regulatory agencies [13]. The multi-residue analysis is mainly based on liquid chromatography [14], such as liquid chromatography-diode array detector (LC-DAD) [15], liquid chromatography-mass spectrometry (LC-MS) [16], liquid chromatography coupled with tandem mass spectrometry (LC-MS/MS) [17,18] and liquid chromatography coupled to high-resolution mass spectrometry (LC-HRMS) [19,20]. UPLC-MS/MS offers a powerful technique for separating, identifying, and quantifying trace components in complex sample matrixes. Thus, various researchers have employed this technique for screening and quantifying antimicrobials in various food matrices [21]. However, there are relatively few reports on Western-style pork products [22].

Due to the complexity of food matrices, the sample pre-treatment process plays a leading role in analyzing antibacterial drug residues [23]. Most of the procedures were time-consuming and tedious, involving solid-phase extraction [20,24], pressurized liquid extraction [25], ultrasound-assisted extraction [26], microwave-assisted extraction [27], and matrix solid-phase dispersion [28]. To simplify the sample treatment procedure, the QuEChERS (acronym of quick, easy, cheap, effective, rugged, safe) approach could be the priority selection because it is easy to operate, has no auxiliary equipment, has low organic solvent consumption, and reduces time [29]. The original QuEChERS method was developed by Anastassiades and coworkers, mainly employed for pesticide residues in non-fatty food samples such as fruits and vegetables [30]. In addition, it is a flexible method that permits modifications depending on analytes and matrices and has extended into different fields, especially for veterinary drug residues [31]. Several modifications have been proposed to improve extraction efficiency, including the addition of organic acids into the extraction phase, as proposed by Stubbings [32], and the use of other sorbents such as C18, NH_2_, and Z-Sep^+^, thus increasing the removal efficiency of pigments, fatty acids, and lipids [33]. Recently, different QuEChERS procedures have been applied in the multi-residue analysis of veterinary drugs in milk, eggs, poultry, fish, and meat [34,35,36]. However, few studies have used the QuEChERS method to optimize the detection of multiple antimicrobial residues in complex matrices such as Western-style meat products.

This work aims to optimize and develop a fast and reliable method based on modified QuEChERS and UPLC-MS/MS to determine 43 antibacterial drugs in bacon and ham. In order to extract all target analytes and efficiently remove interfering substances, the modified QuEChERS program is optimized by multivariate optimization techniques. Single-factor experiments were conducted to investigate several critical parameters, including the type of extraction solvent, acidity, freezing time, and extraction salt. The purification conditions were optimized by Box–Behnken design, and the residues of quinolones and sulfonamides in bacon and ham were successfully analyzed.

## 2. Results and Discussion

After applying the QuEChERS method to extract the target antimicrobial drugs from samples, analysis was performed by UPLC-MS/MS. To obtain good analytical performance, the instrument parameters and the extraction and purification conditions were optimized for sample pre-treatment, respectively.

### 2.1. Optimization of UPLC-MS/MS Analysis

**Figure 1:**
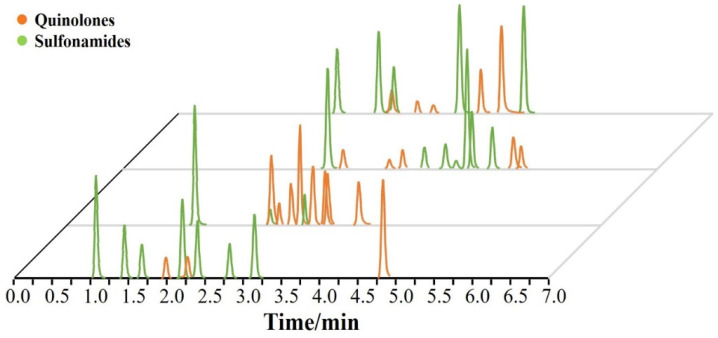
Extracted ion chromatograms of antimicrobial drugs obtained from the spiked sample at 50 µg/kg.

The mass spectrometry of target antimicrobial drugs was determined by direct injection of 200 mg/L analytes in ESI positive ionization mode to optimize the mass spectrometry conditions. There were enough sampling points for each peak to select the most abundant ion as the quantitative and another suitable ion as the qualitative ion. Then, cone voltage and collision energy parameters were optimized to enhance the intensity of the parent ion, and the product ions of each analyte to be measured can be maximized. The desolvation temperature, flow rate, and UPLC gradient elution procedure were also optimized to improve the sensitivity of mass spectrometry systems. Table S1 lists the optimized mass spectrometry parameters and the respective retention times of the antimicrobial drugs, separating the target antimicrobial drugs (quinolones and sulfonamides) within a reasonable time frame. Figure 1 shows the extracted ion chromatogram of the analyzed antimicrobial drugs. 

### 2.2. Optimization of QuEChERS Procedure

Due to the presence of matrix interference, the qualitative identification process requires optimization of the QuEChERS method, which is the most challenging situation for qualitative identification and requires quantitative validation. Several factors that could affect the extraction efficiency of the QuEChERS method were studied through a single-factor experiment. The QuEChERS method was optimized by matrix addition of quinolones and sulfonamides at the level of 50 µg/kg as follows.

#### 2.2.1. Optimization of Extraction Condition

In this experiment, we examined four factors influencing extraction efficiency: the type of extraction solvent, FA volume ratio, freezing time, and extraction salt. Each factor was performed in triplicate in the optimization process. According to Commission Decision No 2002/657/EC, the recommended recoveries are between 80% and 110% for samples spiked above 10 µg/kg. The number of antimicrobial drugs fulfilling the above requirements was selected to assess the effect of different factors on extraction efficiency.

**Figure 2:**
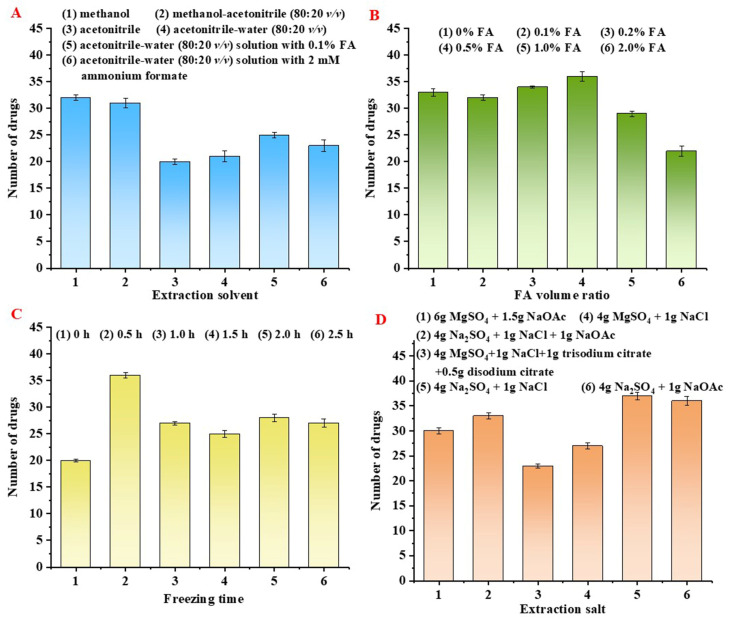
Effects of different extraction factors. (**A**) The effects of different extraction solvents; (**B**) The effect of the acidity of the extraction solvent; (**C**) The effect of freezing time; (**D**) The effect of extraction salt.

Compared with different extraction solvents under the same conditions of extraction efficiency: (1) methanol, (2) methanol-acetonitrile (80:20 *v/v*), (3) acetonitrile, (4) acetonitrile-water (80:20 *v/v*), (5) acetonitrile-water (80:20 *v/v*) solution with 0.1% FA; (6) acetonitrile-water (80:20 *v/v*) solution with 2 mM ammonium formate [5]. As shown in Figure 2A, when the extraction solution was methanol, the highest number of antimicrobial drugs fulfilled the requirement, followed by methanol-acetonitrile (80:20 *v/v*) and acetonitrile-water (80:20 *v/v*) solution of 0.1% FA. Using methanol as the extraction solvent significantly improved the recovery of some sulfonamide antimicrobials with good RSD values. Moreover, the acidity of the extraction solution also affected experimental results. In acidic conditions, the compounds were protonated and increased their solubility [33]. In this sense, the acidity of the extraction solvent was adjusted to 0, 0.1, 0.2, 0.5, 1.0, and 2.0% (*v/v*, adjusted by FA, No. 1−6). As shown in Figure 2B, the best extraction efficiency was obtained when the addition of formic acid was 0.5%. When antimicrobial drugs were present in a low-acidity sample, their ionization was reduced, and the ion exchange with the sorbent was also affected. Nevertheless, lower extraction efficiency at 2.0% FA-methanol could be related to the enhanced matrix effect. Therefore, 0.5% FA-methanol was selected as an extraction solvent for further research.

Some interfering substances were extracted together with the target analytes as co-extracts during the extraction process, such as lipids and proteins, which could be removed by using low-temperature precipitation. This method can be simple without using specialized equipment [37]. Bacon and ham are mainly composed of animal fat and proteins. Therefore, this experiment used a low temperature (−20 °C) precipitation method to reduce interference from co-extracts. As shown in Figure 2C, the freezing time was optimized from 0 h to 2.5 h, corresponding to experimental serial numbers 1−6. The results showed that room temperature (0 h) was insufficient to precipitate the interferences, but too much time (2.5 h) would cause decreased recovery of some drugs. Therefore, the final choice of freezing time of 0.5 h for this experiment. In contrast to previous studies, which required very long freezing times, such as 3 h [38] or 12 h [39], the present experiment only needed 0.5 h to achieve precipitation of the co-extracts.

In the QuEChERS method, salting agents play an essential role. To assess the extraction salt, the various compositions of salt pockets from the methods of published literature were compared [33], including (1) 6 g MgSO_4_ + 1.5 g NaOAc, (2) 4 g Na_2_SO_4_ + 1g NaCl + 1 g NaOAc, (3) 4 g MgSO_4_ + 1 g NaCl + 1 g trisodium citrate + 0.5 g disodium citrate, (4) 4 g MgSO_4_ + 1 g NaCl, (5) 4 g Na_2_SO_4_ + 1g NaCl, (6) 4 g Na_2_SO_4_ + 1 g NaOAc. As shown in Figure 2D, the combination of NaCl or NaOAc with Na_2_SO_4_ showed good extraction efficiency. This result was due to the exothermic hydration reaction that may degrade some antimicrobial drugs after adding anhydrous magnesium sulfate. It is also possible that the interaction between antimicrobial drugs and Mg^2+^ hinders the extraction process [36]. By further experimental comparison, samples containing sodium acetate salt packets treated with nitrogen blowing re-solubilization process required nearly one hour, far beyond the concept of rapid sample pre-treatment, so the 4 g Na_2_SO_4_ + 1 g NaCl salt pocket was selected.

#### 2.2.2. Optimization of Clean-Up Condition

The clean-up step is the key to ensuring the reduction of matrix co-extractants, so there is an urgent need to select suitable clean-up packing materials. Considering Western-style pork products are rich in fat, protein, pigment, and other impurities, PSA, C18, and Z-Sep^+^ were selected as the purification adsorbents for d-SPE in this experiment. PSA can effectively adsorb polar molecules and eliminate polar impurities such as fatty acids, sugars, and pigments. C18 can effectively remove non-polar impurities such as fat-soluble impurities. Z-Sep^+^ can remove pigments and sterols [31]. The effect of the adsorbent on sample recovery and RSD during the pre-treatment was assessed based on the number of compliant antimicrobial drugs at the same concentration. To obtain optimal experimental conditions, the interactions between all factors must be considered [40]. A three-factor, three-level response surface experiment was designed using the Box–Behnken design (BBD). The research used Design Expert software (version 8.0) to design experiments, data analysis, and methodological modeling.

The focus of this experiment was to determine the influence of individual factors in extracting antimicrobial drugs from samples. Three independent variables were selected, C18 (A), PSA (B), and Z-Sep^+^ (C). Moreover, the number of compliant antimicrobial drugs is the dependent variable (response value, Y), all other conditions being equal. Table S2 shows the detailed experimental design, the response surface experiments’ outcome, and the model’s theoretical value. A multiple regression fit was performed using the data in Table S2, and there were the multiple regression equations obtained from Design Expert software: Y = 41.80–0.50A–1.50B–2.75C–0.50AB + 1.00AC + 0.00BC–7.90A^2^–8.40B^2^–2.90C^2^.

The meaning of the regression coefficients was evaluated by ANOVA of the experimental data by the corresponding *p*-values. As shown in Table 1, the *F*-value of the model was 50.78, with only a 0.01% probability that the corresponding *F*-value was because of noise, indicating that this model was significant; the *p*-value < 0.01 indicates that the model was highly significant; the lack-of-fit errors had a *p*-value of 0.1051, showed insignificantly and no lack-of-fit condition. These parameters from the equations showed that the model fits well, the results were significant, and had a small error from the experimental predictions, indicating reproducible data: correlation coefficient *R*^2^ = 0.9849, predicted *R*^2^ = 0.8126, the difference between *R*^2^ and Adj-*R*^2^ < 0.1, Adj-*R^2^* differed from predicted *R*^2^ by <0.2, S/N ratio (Adeq Precision) = 19.293 > 4. The above values indicated that the regression equation was available for analyzing the experimental results. The model had a very high Adj-*R*^2^ = 0.9655, indicating that the model explained 3.45% of the variation in the model. These results showed that in a good performance, the equation reflected three factors that influence the extraction of antimicrobial drugs from samples, with factors B, C, A^2^, B^2^, and C^2^ all expressed very significant (*p* < 0.05), and the sequence of the three factors influenced in extracting antimicrobial drugs was as follows: C > B > A.

**Figure 3:**
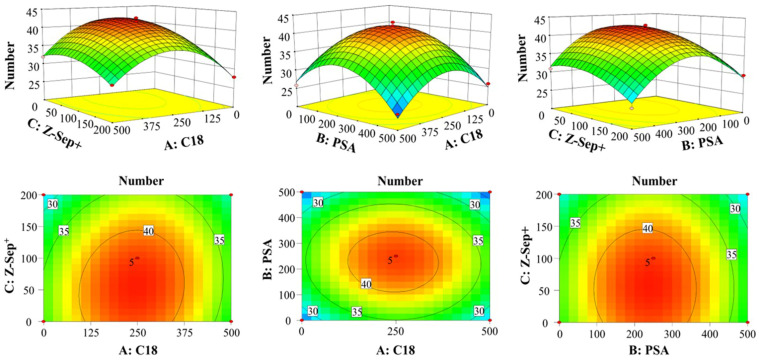
Response surface map for the interaction of various factors.

The 3D response surface of antimicrobial drug extraction interaction in the sample by C18, PSA, and Z-Sep^+^ was shown in Figure 3. Three-dimensional surface plots of the response surface formed by the number of antimicrobial drugs extracted from samples under each influencing factor reflect the optimal extraction terms and the interactions between different factors. If there is a steeper curve, this factor has a more significant effect in extracting antimicrobial drugs. As seen in Figure 3, Z-Sep^+^ had the most significant effect on the experimental results, followed by PSA, and C18 had the slightest effect. The most significant interaction was between C18 and Z-Sep^+^, followed by C18 and PSA, and the minor interaction was between PSA and Z-Sep^+^.

The best experimental conditions were determined based on the response surface analysis results: C18 235.2 mg, PSA 228.2 mg, and Z-Sep^+^ 51.5 mg. Under these conditions, the theoretical prediction of the number of detected antimicrobial drugs is 42. Five parallel experiments were conducted to verify the optimal extraction conditions. The average and relative standard deviation of the number of antimicrobial drugs were 42.8 and 0.9%, respectively, which showed that the regression model predictions were accurate, reliable, and reproducible.

### 2.3. Method Performance and Validation

The proposed method was validated under the optimal experimental conditions according to the standards set by the European Community Decision 2002/657/EC on CCα, CCβ, precision, accuracy, and selectivity. Linearity, LOQ, and matrix effect were also determined.

#### 2.3.1. Matrix Effect

Impurities such as fats, lipids, and proteins in bacon and ham can inhibit or enhance the response to antimicrobial drugs. The matrix effect is calculated as follows:

$$\mathrm{ME}\;\left(\%\right)=\left(\frac{slope\;of\;the\;matrix-matched\;standard}{slope\;of\;the\;solvent\;standard}-1\right)\times 100$$

**Figure 4:**
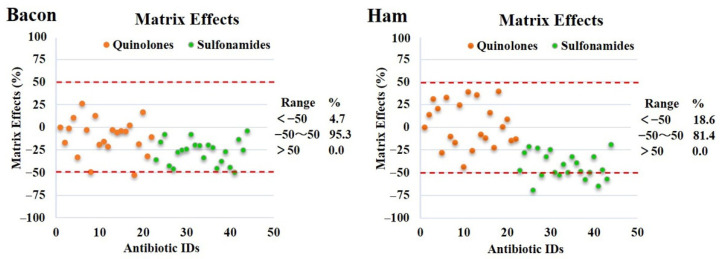
Matrix effect distribution of antimicrobial drugs in bacon and ham.

The effect is mild or medium when ME ranges from −50% to 50% and strong when ME is lower than −50% or greater than 50% [39]. As shown in Figure 4, more than 81.4% of the antimicrobial drugs showed mild or moderate matrix effects in both bacon and ham. The proportion of antimicrobial drugs with ME < −50 was 4.7% and 18.6% in bacon and ham, respectively. As matrix effects could not be wholly excluded from the sample, there were ways to mitigate matrix effects, of which matrix-matched calibration curves seem to be the best solution. 

#### 2.3.2. Selectivity and Linearity

The method was based on UPLC-MS/MS to assess the selectivity of the two types of Western-style pork products. In order to reduce the differences in matrix composition, the blank and commercially available samples were the same types of samples. The signals of the target analytes in blank and spiked samples were compared at the same retention time. No signals of the target analytes were observed in the blank samples, indicating that the method is selective. In addition, the interfering compounds caused no significant interference with the retention time of the target analytes.

The linear range of the proposed method was assessed using matrix-matched calibration curves. Blank samples were prepared following the optimized modified QuEChERS method, and all experiments were determined three times in parallel, using previously configured standard stock solutions. Good linearity was achieved in the dynamic range of 0.01–100 μg/L (*R*^2^ > 0.99), and the detailed results are shown in Table S3.

#### 2.3.3. CCα, CCβ, and LOQ

The stability experiments showed that the selected factors had no significant effect on the analytical results. As Table 2 shown, CCα ranged from 10.9–31.3 μg/kg, and CCβ ranged from 11.8–52.5 μg/kg. Except for CCα and CCβ, LOQ is the minimum additional amount at a signal-to-noise ratio of 10:1, and LOQ ranges from 0.05 to 5 μg/kg. Currently, the maximum residue limits (MRLs) were not set for quinolones and sulfonamides in bacon and ham, but MRLs for these drugs had been set for pork, which is the main component of bacon and ham (all >50 μg/kg). Therefore, this method was sensitive enough to monitor antimicrobial drugs in Western-style pork products.

#### 2.3.4. Accuracy and Precision

To determine how accurate and precise the method is, six parallel determinations were performed at three spiked levels of 5, 10, and 50 μg/kg to calculate the recoveries (Rec, %) and relative standard deviations (RSD, %) for the same day (intra-day precision) and three consecutive days (inter-day precision). All antimicrobial drug recovery results were shown in Table S4, which showed acceptable recoveries (71.2–119.6%) and relative standard deviations (RSD ≤ 19.1%) for all analytes. The accuracy of the method was determined by repeatability and reproducibility experiments. In Table S4, the RSD expressed as intra-day and inter-day precision were below 10.7% and 18%, respectively. The above results demonstrate that the proposed method was feasible for determining antimicrobial drug residues in Western-style pork products.

### 2.4. Analysis of Actual Samples

**Figure 5:**
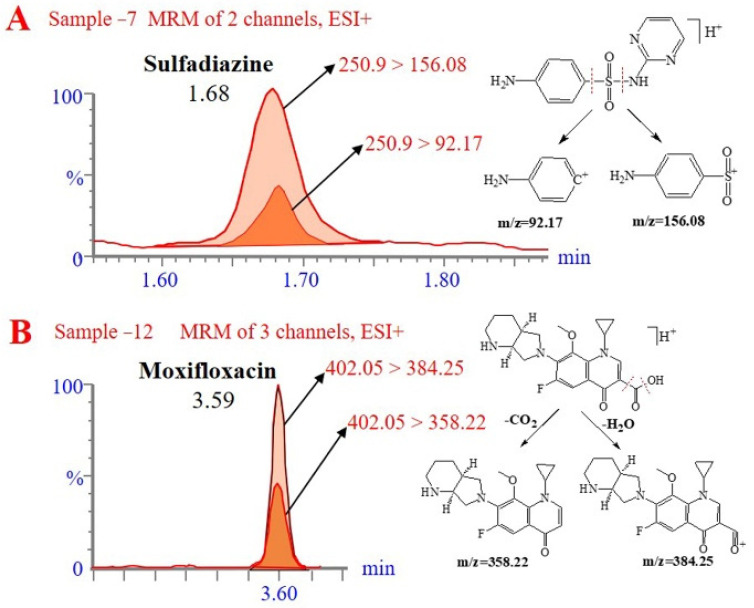
Example chromatograms of antimicrobial drugs (at μg/kg) detected in samples. (**A**) Sulfadiazine at 0.6 μg/kg in sample 7 (ham); (**B**) moxifloxacin at 10.9 μg/kg in sample 12 (bacon).

The established method tested 28 actual samples, including bacon (14 samples) and ham (14 samples) from local supermarkets. Sulfadiazine and moxifloxacin were detected in sample 7 and sample 12, respectively, and the concentrations of antimicrobial drugs detected were 0.6 and 10.9 μg/kg. The remaining samples had no antimicrobial drugs detected. The above-detected drugs were likely used to treat or prevent disease during breeding. Figure 5 shows the chromatograms of the sulfadiazine and moxifloxacin-positive samples.

## 3. Materials and Methods

### 3.1. Chemicals and Reagents

All antimicrobial drug standards (≥97% purities) were obtained from Alta (Tianjin, China). MS-grade methanol, acetonitrile, formic acid (FA), ammonium formate, and HPLC-grade toluene were obtained from Fisher (Branchburg, NJ, USA). Analytical grade anhydrous Na_2_SO_4_, anhydrous MgSO_4_, trisodium citrate, disodium citrate, sodium chloride (NaCl), and sodium acetate (NaOAc) were purchased from Beijing Chemical Plant (Beijing, China); A bonded C18 zirconia-coated silica (Z-Sep^+^) was obtained from Sigma (Osterode am Harz, Germany). Primary secondary amine (PSA) and octadecyl silane (C18) were obtained from Agilent Technologies (Santa Clara, CA, USA). Ultra-pure water was prepared by Milli-Q ultrapure water machine obtained from Millipore Corporation (Burlington, MA, USA).

Forty-three antimicrobial drug standards are listed in Table S1, including quinolones and sulfonamides. Usually, individual stock solutions of drugs were prepared in methanol or acetonitrile at 500−1000 mg/L concentrations. All stock solutions could be stored stably for six months in closed brown-colored volumetric flasks under −20 °C refrigeration conditions. The standard working solution was diluted with methanol to the desired concentration (stored at −20 °C and prepared once a month).

### 3.2. Instrumentation Parameters

The ultra-performance liquid chromatography coupled with tandem mass spectrometry consisted of the ACQUITY™ I-Class UPLC™ system (Waters Corporation, Milford, MA, USA) in conjunction with Xevo™ TQ-S mass spectrometer (Waters Corporation, Milford, MA, USA). The optimal chromatographic separation conditions were provided as follows: reversed-phase chromatography column (ACQUITY UPLC BEH T3 C18 column 2.1 mm × 50 mm, 1.7 µm; Waters Corporation, Milford, MA, USA); mobile phase A was water containing 0.1% formic acid; mobile phase B was 0.1% formic acid acetonitrile/methanol (80:20 *v/v*); gradient elution program, 0 min: 5% B, 2 min: 15% B, 5 min: 40% B, 7 min: 95% B, 7.1 min: 5% B, run after 2 min. The injection volume, flow rate, and column temperature were set at 2 μL, 0.3 mL/min, and 35 °C, respectively. 

The optimal mass spectrometry conditions were set as follows: the scan mode was MRM (multiple reaction monitoring); capillary voltage was 3 kV; nebulizer gas was 7 Bar; desolvation temperature at 550 °C with a flow rate of 1100 L/Hr; cone voltage was set at 51 V with flow rate 150 L/Hr; collision gas flow was 0.15 mL/Min.

### 3.3. Sample Preparation

To obtain representative information on antimicrobial residues in Western-style pork products, we purchased 28 samples from different local supermarkets, including bacon and ham, to cover as many other brands as possible and stored them at 4 °C. Each sample was homogenized with an electric meat grinder and stored at −20 °C.

The QuEChERS procedure was as follows: 50 mL centrifuge tube containing 2.0× *g* of homogenized sample. Twenty milliliters of 0.5% FA-methanol (*v/v*) was added, vortexed for 0.5 min (vortex mixer, AS ONE, Osaka, Japan), followed by extraction salt pack (4 g Na_2_SO_4_, 1g NaCl), and shaken for 2 min. Then, the samples were kept in the dark at −20 °C for 0.5 h before centrifuging at 10,000× *g* rpm for 5 min (high-speed centrifugation, Sigma, Osterode am Harz, Germany), transferred 10 mL of supernatant to a 15 mL purification tube (containing 235.2 mg C18, 228.2 mg PSA, and 51.5 mg Z-Sep^+^). The clean-up tube was shaken for 2 min, centrifuged at 4200× *g* rpm for 5 min (low-speed centrifuge, Zonkia, Hefei, China), and then 2 mL of supernatant was transferred to a 10 mL glass tube in a 35 °C water bath and evaporated to dryness with a gentle stream of nitrogen (nitrogen-blowing concentrator, Organomation Associates, Berlin, MA, USA). At last, it was re-dissolved with 1 mL of acetonitrile/water (1:9 *v/v*) solution, filtered through a 0.22 μm PTFE membrane, before being analyzed by UPLC-MS/MS.

### 3.4. Validation of the Method

The developed method was validated following the recommendations of the validation guides provided by the European Community Decision 2002/657/EC [41]. The performance parameters to be evaluated during method validation were decision limit (CCα), detection capability (CCβ), selectivity, recovery (accuracy), intra-day precision (repeatability), and inter-day precision (reproducibility). Linearity, LOQ, and matrix effect (ME) were also determined.

The method selectivity was verified by determining 20 blank samples (including different types: bacon and ham) to determine that there were no potential interferents with the same retention time as the target compounds. 

The validation levels (VL) of the antimicrobial drugs were chosen below the MRL values to prevent the overloading of the chromatography column. The primary source of samples is pork, for which the European Commission or China set MRLs of 200 µg/kg for most antimicrobial drugs (quinolones and sulfonamides) and 100 µg/kg or less for other antibiotics [11,12]. However, no MRLs have been set for these drugs in Western-style pork products. Thus, we set the VL to 50 μg/kg, considering a combination of detection requirements, major drug residues in the actual sample matrix, and the expected sensitivity limit of UPLC-MS/MS. Therefore, the validation concentrations of the recovery experiments were all set near VL, which were 0.1 × VL, 0.2 × VL, and 1 × VL, respectively.

The precisions of the method were investigated by analyzing the antimicrobial drugs in spiked blank samples. On each validation day, six parallel samples of each sample were injected into the system to assess intra-day variability. The validation procedure was replicated consecutively for three days to determine the inter-day variability of the analytical method.

As bacon and ham do not have the relevant MRLs set, the following calculation method applies. For the analytes without MRL in our validation experiments, the concentration level was set to 0.2 × VL [42]. CCα and CCβ were determined by 0.2 × VL established by the following equations:

CCα = 0.2 × VL + 1.64 × SD_0.2 × VL_, (1)

CCβ = CCα + 1.64 × SD_0.2 × VL_, (2)

where SD_0.2 × VL_ stands for the standard deviation at the 0.2 × VL level

The matrix effect was determined with the following equation:

ME (%) = (S_Mat_/S_Sol_ − 1) × 100, (3)

where S_Mat_ is the slope of the matrix matched standard.

where S_Sol_ is the slope of the solvent standard.

The LOQ calculation formula is as follows:

LOQ = 10 × S/N,(4)

where S/N is the ratio of signal to noise of the target antimicrobial drug at the corresponding retention time in the blank sample matrix.

## 4. Conclusions

This study used a multivariate optimization strategy, developing a multi-residue analytical method based on the modified QuEChERS combined with UPLC-MS/MS to determine 43 antimicrobial drugs in bacon and ham. Box–Behnken response surface design could determine the optimal parameters for experimental factors, effectively reducing the number of experiments, reagent consumption, and time required. All these results showed that the proposed method was valuable for applying and determining multiple antimicrobial drug residues in bacon and ham, and successfully detecting residues of sulfadiazine and moxifloxacin in actual samples. Thus, the proposed method could be reliable and suitable for routine analysis of multiple antimicrobial drug residues in Western-style pork products.

## Figures and Tables

**Table 1:** Variance analysis of regression model of antimicrobial drugs extraction from spiked samples.

Source	Sum of Squares	Degrees of Freedom	Mean Square	*F*-Value	*p*-Value (Prob > *F*)	Distinctiveness
Model	737.76	9	81.97	50.78	<0.0001	significant
A-C18	2	1	2	1.24	0.3024	
B-PSA	18	1	18	11.15	0.0124	*
C-Z-Sep^+^	60.5	1	60.5	37.48	0.0005	**
AB	1	1	1	0.62	0.4571	
AC	4	1	4	2.48	0.1595	
BC	0	1	0	0	1	
A^2^	262.78	1	262.78	162.78	<0.0001	**
B^2^	297.09	1	297.09	184.04	<0.0001	**
C^2^	35.41	1	35.41	21.94	0.0023	*
Residual	11.3	7	1.61			
Lack of fit	8.5	3	2.83	4.05	0.1051	not significant
Pure error	2.8	4	0.7			
Cor total	749.06	16				

Notes: *, *p* < 0.05 means that the difference is significant, **, *p* < 0.01 means that the difference is very significant.

**Table 2:** Validation parameters for the developed UPLC-MS/MS method.

Compound	Bacon			Ham		
	ccα	ccβ	LOQ	ccα	ccβ	LOQ
	(μg/kg)	(μg/kg)	(μg/kg)	(μg/kg)	(μg/kg)	(μg/kg)
**Quinolones (21)**						
cinoxacin	16.2	22.5	0.1	20.2	30.3	0.05
ciprofloxacin	24.7	39.4	2	21.6	33.3	5
danofloxacin	12.4	14.8	0.1	24.4	38.8	0.2
difluoxacin	22.4	34.8	1	18.9	27.8	1
enoxacin	16.4	22.9	0.5	26.3	42.6	0.1
enrofloxacin	25.2	40.3	2	20.2	30.5	0.5
fleroxacin	24.9	39.8	2	21.7	33.4	10
flumequine	15.5	21	0.1	19.4	28.7	0.05
gatifloxacin	20.5	31	0.2	27.6	45.2	1
gemifioxacin	13.9	17.8	0.1	23.3	36.6	0.2
lomefloxacin	20.9	31.8	1	23.3	36.6	5
marbofloxacin	13.2	16.4	0.5	27.1	44.1	0.5
moxifloxacin	11.8	13.7	1	31.3	52.5	0.5
nadifloxacin	14.6	19.2	0.1	21.1	32.1	0.1
nalidixic acid	15.1	20.2	0.2	17.3	24.6	0.1
ofloxacin	25.9	41.8	2	27.1	44.2	5
orbifloxacin	23.8	37.7	5	29.4	48.8	2
oxolinic acid	18	26	0.1	19.7	29.3	0.1
pefloxacin	14.9	19.7	0.1	24.0	38.0	0.5
sarafloxacin	12.7	15.4	2	25.9	41.8	1
sparfloxacin	25.7	41.3	1	23.2	36.3	1
**Sulfonamides (22)**						
sulfabenzamide	15.4	20.8	0.05	24.7	39.5	0.1
sulfachloropyridazine	17.8	25.6	1	25.1	40.1	2
sulfaclozine	18.8	27.6	0.1	26.7	43.5	1
sulfadiazine	11.6	13.3	0.05	16.5	23.0	0.1
sulfadimidine	16.2	22.5	0.1	22.7	35.3	0.05
sulfadoxine	12.8	15.6	0.1	24.6	39.3	0.05
sulfamerazine	13.9	17.8	0.2	18.4	26.8	1
sulfameter	11.9	13.9	0.2	25.7	41.5	1
sulfamethizole	17.3	24.5	0.1	18.1	26.1	0.5
sulfamethoxazole	14.2	18.4	0.05	20.6	31.3	0.05
sulfamethoxypyridazine	16	22	0.1	24.6	39.2	0.2
sulfamonomethoxine	21.8	33.6	0.1	21.4	32.7	0.2
sulfamoxole	19.4	28.9	0.1	21.6	33.2	0.2
sulfaphenazole	12.1	14.1	0.05	20.6	31.1	0.05
sulfapyrazole	12.7	15.3	0.1	16.8	23.7	0.05
sulfapyridine	15.5	20.9	0.05	19.2	28.3	0.2
sulfaquinoxaline	16.3	22.5	0.05	22.1	34.2	0.05
sulfathiazole	10.9	11.8	0.1	18.1	26.1	0.05
sulfisomidine	13.4	16.9	0.05	17.0	24.1	0.1
sulfisoxazole	15.6	21.2	0.05	18.8	27.6	0.05
sulfadimethoxine	13.5	16.9	0.05	13.7	17.4	0.05
trimethoprim	13.7	17.3	0.05	20.7	31.4	0.5

## Data Availability

Not applicable.

## Supplementary Materials

The following supporting information can be downloaded at: https://www.mdpi.com/article/10.3390/molecules27238283/s1, Table S1: UPLC-MSMS parameters of the forty-three analytes; Table S2: Design and results of response surface test; Table S3: Validation parameters for linearity; Table S4: Evaluation of accuracy and precision for the developed method in bacon and ham spiked at 5, 10 and 50 µg/kg.

## Sample Availability

Samples of the compounds are not available from the authors.
