# Supplementary material for: Development and Validation of a Method for Determination of 43 Antimicrobial Drugs in Western-Style Pork Products by UPLC-MS/MS with the Aid of Experimental Design
Source: Molecules. 2022 Nov 28;27(23):8283. doi: 10.3390/molecules27238283 (PMC9739473; doi:10.3390/molecules27238283)
Supplement: Supplementary file 1 [file molecules-27-08283-s001.zip › molecules-2041364-supplementary.pdf]

Table S1 UPLC-MSMS parameters of the forty-three analytes.

Compound	Formula	Precursor ion	RT (min)	Cone voltage (V)	MRM 1 (Quantification)	Collision energy 1 (eV)	MRM 2 (Confirmation)	Collision energy 2 (eV)
**Quinolones（21）**								
cinoxacin	$C_{12}H_{10}N_2O_5$	$[M+H]^+$	3.91	23	262.86 > 245.15	24	262.86 > 217.14	36
ciprofloxacin	$C_{17}H_{18}FN_3O_3$	$[M+H]^+$	2.93	95	332.00 > 288.24	26	332.00 > 245.21	38
danofloxacin	$C_{19}H_{20}FN_3O_3$	$[M+H]^+$	3.17	85	358.04 > 340.34	30	358.04 > 96.16	42
difluoxacin	$C_{21}H_{19}F_2N_3O_3$	$[M+H]^+$	3.63	90	400.04 > 382.24	38	400.04 > 356.31	32
enoxacin	$C_{15}H_{17}FN_4O_3$	$[M+H]^+$	2.65	95	320.99 > 303.20	28	320.99 > 205.87	42
enrofloxacin	$C_{19}H_{22}FN_3O_3$	$[M+H]^+$	3.25	85	360.07 > 316.30	30	360.07 > 245.17	40
fleroxacin	$C_{17}H_{18}F_3N_3O_3$	$[M+H]^+$	0.09	90	370.07 > 326.32	30	370.07 > 269.18	42
flumequine	$C_{14}H_{12}FNO_3$	$[M+H]^+$	0.09	38	261.95 > 244.13	28	261.95 > 202.10	54
gatifloxacin	$C_{19}H_{22}FN_3O_4$	$[M+H]^+$	3.47	85	376.06 > 332.25	26	376.06 > 261.20	50
gemifioxacin	$C_{18}H_{20}FN_5O_4$	$[M+H]^+$	3.95	53	390.05 > 372.30	28	390.05 > 313.25	44
lomefloxacin	$C_{17}H_9F_2N_3O_3$	$[M+H]^+$	3.09	90	352.07 > 308.31	26	352.07 > 265.21	36
marbofloxacin	$C_{17}H_{19}FN_4O_4$	$[M+H]^+$	2.57	85	363.08 > 72.16	38	363.08 > 320.21	24
moxifloxacin	$C_{21}H_{24}FN_3O_4$	$[M+H]^+$	3.70	40	402.05 > 358.22	20	402.05 > 384.25	22
nadifloxacin	$C_{19}H_{21}FN_2O_4$	$[M+H]^+$	5.24	53	361.06 > 343.29	38	361.06 > 342.96	56
nalidixic acid	$C_{12}H_{12}N_2O_3$	$[M+H]^+$	5.12	38	232.95 > 215.12	22	232.95 > 104.04	60
ofloxacin	$C_{18}H_{20}FN_3O_4$	$[M+H]^+$	2.81	85	362.08 > 318.33	30	362.08 > 261.18	46
orbifloxacin	$C_{19}H_{20}F_3N_3O_3$	$[M+H]^+$	3.29	95	396.04 > 295.25	36	396.04 > 352.34	28
oxolinic acid	$C_{13}H_{11}NO_5$	$[M+H]^+$	4.24	65	261.94 > 244.17	28	261.94 > 216.16	48
pefloxacin	$C_{17}H_{20}FN_3O3$	$[M+H]^+$	2.85	90	334.08 > 316.26	28	334.08 > 290.25	26
sarafloxacin	$C_{20}H_{17}F_2N_3O_3$	$[M+H]^+$	3.53	90	386.06 > 342.26	30	386.06 > 299.23	44
sparfloxacin	$C_{19}H_{22}F_2N_4O_3$	$[M+H]^+$	3.63	85	393.03 > 393.25	20	393.03 > 349.37	34
**Sulfonamides（22）**								
sulfabenzamide	$C_{13}H_{12}N_2O_3S$	$[M+H]^+$	4.21	55	276.90 > 156.04	17	276.90 > 92.04	38
sulfachloropyridazine	$C_{10}H_9ClN_4O_2S$	$[M+H]^+$	3.38	70	284.97 > 156.02	22	284.97 > 92.13	46
sulfaclozine	$C_{10}H_9ClN_4O_2S$	$[M+H]^+$	4.35	75	284.90 > 156.01	20	284.90 > 92.10	42
sulfadiazine	$C_{10}H_{10}N_4O_2S$	$[M+H]^+$	1.65	23	250.90 > 156.08	15	250.90 > 92.17	28
sulfadimidine	$C_{12}H_{14}N_4O_2S$	$[M+H]^+$	2.71	53	278.97 > 186.08	24	278.97 > 156.09	30
sulfadoxine	$C_{12}H_{14}N_4O_4S$	$[M+H]^+$	3.67	53	310.92 > 156.09	30	310.92 > 92.11	48
sulfamerazine	$C_{11}H_{12}N_4O_2S$	$[M+H]^+$	2.18	48	264.95 > 156.05	28	264.95 > 92.07	44
sulfameter	$C_{11}H_{12}N_4O_3S$	$[M+H]^+$	2.79	53	280.89 > 156.08	26	280.89 > 92.09	44
sulfamethizole	$C_9H_{10}N_4O_2S_2$	$[M+H]^+$	2.84	55	270.88 > 156.04	20	270.88 > 92.08	40
sulfamethoxazole	$C_{10}H_{11}N_3O_3S$	$[M+H]^+$	3.66	48	253.89 > 156.05	22	253.89 > 92.10	40
sulfamethoxypyridazine	$C_{11}H_{12}N_4O_3S$	$[M+H]^+$	2.91	53	280.89 > 156.11	26	280.89 > 92.13	42
sulfamonnomethoxine	$C_{11}H_{12}N_4O_3S$	$[M+H]^+$	3.32	53	280.89 > 156.07	26	280.89 > 92.15	42
sulfamoxole	$C_{11}H_{13}N_3O_3S$	$[M+H]^+$	3.93	48	267.95 > 156.03	22	267.95 > 92.12	46

sulfaphenazole	$C_{15}H_{14}N_4O_2S$	$[M+H]^+$	4.52	65	315.01 > 158.19	40	315.01 > 92.09	56
sulfapyrazole	$C_{16}H_{16}N_4O_2S$	$[M+H]^+$	4.84	53	328.99 > 172.17	44	328.99 > 92.16	56
sulfapyridine	$C_{11}H_{11}N_3O_2S$	$[M+H]^+$	2.04	48	249.95 > 156.05	26	249.95 > 92.13	44
sulfaquinoxaline	$C_{14}H_{12}N_4O_2S$	$[M+H]^+$	4.57	53	300.91 > 156.08	26	300.91 > 92.11	44
sulfathiazole	$C_9H_9N_3O_2S_2$	$[M+H]^+$	1.96	48	255.93 > 156.04	22	255.93 > 92.12	44
sulfisomidine	$C_{12}H_{14}N_4O_2S$	$[M+H]^+$	1.59	58	278.97 > 124.16	34	278.97 > 92.14	52
sulfisoxazole	$C_{11}H_{13}N_3O_3S$	$[M+H]^+$	2.64	50	267.95 > 156.05	20	267.95 > 113.14	22
sulfadimethoxine	$C_{12}H_{14}N_4O_4S$	$[M+H]^+$	4.50	53	310.90 > 156.10	28	310.90 > 92.10	48
trimethoprim	$C_{14}H_{18}N_4O_3$	$[M+H]^+$	2.62	75	291.03 > 123.09	40	291.03 > 230.22	40

Table S2. Design and results of response surface test.

Test Number	Factor			The number of detected antimicrobial drugs
	A:C18 (mg)	B: PSA (mg)	C: Z-Sep$^+$ (mg)	
1	250	250	100	42
2	500	0	100	22
3	500	250	0	32
4	250	500	200	25
5	250	500	0	31
6	250	250	100	41
7	250	250	100	43
8	0	250	200	28
9	0	500	100	30
10	0	0	100	26
11	250	250	100	43
12	0	250	0	35
13	500	500	100	24
14	250	0	200	30
15	250	250	100	39
16	500	250	200	29
17	250	0	0	36

Table S3. Validation parameters for linearity.

Compound	bacon		ham	
	R^2	Linear Range (μg/L)	R^2	Linear Range (μg/L)
**Quinolones（21）**				
cinoxacin	0.9906	0.02～20	0.9981	0.01 ~ 20
ciprofloxacin	0.9960	0.4～100	0.9949	1 ~ 100
danofloxacin	0.9916	0.02～20	0.9945	0.04 ~ 20
difluoxacin	0.9904	0.2～20	0.9959	0.2 ~ 20

enoxacin	0.9914	0.1～20	0.9934	0.02 ~ 20
enrofloxacin	0.9968	0.4～20	0.9967	0.1 ~ 20
fleroxacin	0.9955	0.4～100	0.9923	2 ~ 100
flumequine	0.9982	0.02～20	0.9988	0.01 ~ 20
gatifloxacin	0.9908	0.04～20	0.9921	0.2 ~ 20
gemifioxacin	0.9953	0.02～20	0.9980	0.04 ~ 20
lomefloxacin	0.9906	0.2～100	0.9987	1 ~ 100
marbofloxacin	0.9925	0.1～20	0.9984	0.1 ~ 20
moxifloxacin	0.9913	0.2～20	0.9955	0.1 ~ 100
nadifloxacin	0.9969	0.02～20	0.9983	0.02 ~ 20
nalidixic acid	0.9962	0.04～20	0.9984	0.02 ~ 20
ofloxacin	0.9925	0.4～100	0.9901	1 ~ 100
orbifloxacin	0.9902	1～100	0.9947	0.4 ~ 100
oxolinic acid	0.9989	0.02～20	0.9982	0.02 ~ 20
pefloxacin	0.9927	0.02～20	0.9951	0.1 ~ 20
sarafloxacin	0.9919	0.4～100	0.9955	0.2 ~ 100
sparfloxacin	0.9989	0.2～20	0.9997	0.2 ~ 100
**Sulfonamides（22）**				
sulfabenzamide	0.9988	0.01～20	0.9996	0.02 ~ 20
sulfachloropyridazine	0.9942	0.2～20	0.9950	0.4 ~ 100
sulfaclozine	0.996	0.02～20	0.9962	0.2 ~ 20
sulfadiazine	0.9984	0.01～20	0.9988	0.02 ~ 20
sulfadimidine	0.9972	0.02～20	0.9959	0.01 ~ 20
sulfadoxine	0.9962	0.02～20	0.9968	0.01 ~ 20
sulfamerazine	0.9966	0.04～20	0.9901	0.2 ~ 100
sulfameter	0.9985	0.04～20	0.9909	0.2 ~ 20
sulfamethizole	0.9950	0.02～20	0.9989	0.1 ~ 20
sulfamethoxazole	0.9968	0.01～20	0.9955	0.01 ~ 20
sulfamethoxypyridazine	0.9963	0.02～20	0.9971	0.04 ~ 20
sulfamonomethoxine	0.9948	0.02～20	0.9982	0.04 ~ 20
sulfamoxole	0.9983	0.02～20	0.9995	0.04 ~ 20
sulfaphenazole	0.9984	0.01～20	0.9988	0.01 ~ 20
sulfapyrazole	0.9982	0.02～20	0.9989	0.01 ~ 20
sulfapyridine	0.9971	0.01～20	0.9978	0.04 ~ 20
sulfaquinoxaline	0.9991	0.01～20	0.9987	0.01 ~ 20
sulfathiazole	0.9993	0.02～20	0.9988	0.01 ~ 20
sulfisomidine	0.9992	0.01～20	0.9997	0.02 ~ 20
sulfisoxazole	0.9970	0.01～20	0.9988	0.01 ~ 20
sulfadimethoxine	0.9996	0.01～20	0.9987	0.01 ~ 20
trimethoprim	0.9904	0.01～40	0.9988	0.1 ~ 20

Table S4. Evaluation of accuracy and precision for the developed method in bacon and ham spiked at 5, 10 and 50 μg/kg.

Compound	bacon								ham							
	5 μg/kg (0.1×VL)		10 μg/kg (0.2×VL)		50 μg/kg (1×VL)		Intra-day (n=6)	Inter-day (n=18)	5 μg/kg (0.1×VL)		10 μg/kg (0.2×VL)		50 μg/kg (1×VL)		Intra-day (n=6)	Inter-day (n=18)
	REC(%)	RSD(%)	REC(%)	RSD(%)	REC(%)	RSD(%)	RSD(%)	RSD (%)	REC(%)	RSD(%)	REC(%)	RSD(%)	REC(%)	RSD(%)	RSD(%)	RSD (%)
**Quinolones （21）**																
cinoxacin	112.3	9.1	104.1	4.7	90.6	4.8	4.7	5.0	107.6	3.1	98.0	3.9	88.6	2.1	5.4	5.9
ciprofloxacin	98.7	8.2	77.4	12.4	82.4	3.5	0.5	13.0	81.9	5.1	83.2	5.5	84.5	5.9	8.1	7.2
danofloxacin	102.9	8.8	105.8	9.6	88.7	9.4	4.1	3.6	100.4	5.2	95.2	4.6	99.9	4.7	5.3	10.6
difluoxacin	104.6	6.6	103.0	8.1	92.1	7.6	6.0	17.6	94.7	10.2	105.3	3.3	105.5	2.9	4.1	4.8
enoxacin	76.3	8.1	108.0	2.1	92.8	8.0	8.0	4.7	76.4	5.4	98.0	5.4	91.5	3.8	5.5	12.2
enrofloxacin	98.3	2.9	96.5	13.7	107.5	8.1	5.5	9.5	102.1	5.3	87.6	4.9	96.4	5.1	8.7	9.6
fleroxacin	110.5	8.2	91.4	7.7	103.5	5.2	1.8	13.5	104.2	4.7	93.8	4.3	109.4	5.1	10.4	15.7
flumequine	92.3	3.1	97.9	8.0	83.6	1.8	0.3	2.8	104.0	5.4	100.5	5.1	94.5	3.8	3.6	6.3
gatifloxacin	94.4	10.9	87.9	12.3	106.6	11.2	3.0	12.2	99.6	4.8	104.3	5.0	97.8	3.4	7.7	10.3
gemifioxacin	99.2	5.6	100.5	10.2	106.9	7.2	5.0	2.0	102.4	4.0	96.4	2.6	101.1	3.8	5.1	16.0
lomefloxacin	113.4	6.8	115.6	11.5	95.6	9.0	5.1	11.5	107.1	9.6	92.9	7.0	93.2	10.0	4.1	5.3
marbofloxacin	111.7	15.7	115.3	8.5	91.8	9.6	0.3	2.6	103.8	8.7	98.1	8.1	98.3	4.2	3.0	8.7
moxifloxacin	97.6	14.8	71.5	5.9	93.6	10.7	1.0	6.0	95.4	6.2	103.7	6.5	98.8	5.0	9.1	6.9
nadifloxacin	106.9	3.5	96.1	13.3	107.0	8.0	0.1	2.8	92.0	9.3	100.5	7.9	89.4	11.1	8.8	9.9
nalidixic acid	94.5	6.3	101.5	5.7	104.6	1.4	2.3	2.8	102.4	5.7	107.5	4.7	97.4	5.9	6.7	11.6
ofloxacin	106.1	19.1	83.5	8.8	103.1	4.1	5.0	7.8	102.7	7.7	99.9	6.6	94.2	3.7	5.8	11.3
orbifloxacin	107.6	13.0	93.4	8.1	108.9	8.7	4.0	10.6	95.1	7.4	97.2	4.7	105.2	5.3	7.2	9.8
oxolinic acid	93.0	8.6	101.5	7.4	109.6	1.7	1.3	4.8	108.3	5.3	104.9	6.8	94.2	5.6	8.0	9.6
pefloxacin	119.6	10.8	114.7	13.8	81.3	9.3	0.2	2.5	96.9	6.7	97.9	4.1	109.3	5.5	7.3	13.7
sarafloxacin	79.4	10.2	102.8	5.5	98.4	9.3	4.3	14.4	101.3	6.4	99.4	5.0	105.9	5.1	9.1	11.1
sparfloxacin	109.3	9.5	109.3	9.5	100.5	8.8	8.9	16.0	113.6	7.9	87.9	4.3	111.6	5.7	10.7	13.8

Continued Table S4. Evaluation of accuracy and precision for the developed method in bacon and ham spiked at 5, 10 and 50 μg/kg.

Compound	bacon								ham							
	5 μg/kg (0.1×VL)		10 μg/kg (0.2×VL)		50 μg/kg (1×VL)		Intra-day (n=6)	Inter-day (n=18)	5 μg/kg (0.1×VL)		10 μg/kg (0.2×VL)		50 μg/kg (1×VL)		Intra-day (n=6)	Inter-day (n=18)
	REC(%)	RSD(%)	REC(%)	RSD(%)	REC(%)	RSD(%)	RSD(%)	RSD (%)	REC(%)	RSD(%)	REC(%)	RSD(%)	REC(%)	RSD(%)	RSD(%)	RSD (%)
**Sulfonamides（22）**																
sulfabenzamide	80.6	8.9	110.7	5.7	84.9	5.2	0.8	2.7	104.8	4.7	99.9	5.8	108.6	5.1	5.0	7.6
sulfachloropyridazine	101.6	10.5	107.4	12.9	83.7	6.1	2.5	10.6	104.7	4.3	99.7	5.2	100.9	5.2	1.6	5.3
sulfaclozine	102.1	17.7	97.0	11.0	99.7	8.8	6.2	4.7	101.6	9.9	99.2	4.1	105.2	4.1	3.4	5.5
sulfadiazine	101.9	4.8	103.3	4.2	98.6	1.7	1.2	1.5	116.0	4.4	101.1	9.3	92.4	4.8	1.6	5.2
sulfadimidine	105.5	7.5	94.5	5.4	88.8	5.0	1.5	3.2	103.0	5.2	93.9	6.1	109.0	5.3	4.9	11.4
sulfadoxine	107.8	4.4	97.3	3.0	102.3	5.0	1.4	3.2	95.2	9.8	97.4	6.4	101.5	4.2	5.4	8.7
sulfamerazine	114.2	3.7	116.8	10.7	88.3	8.4	3.0	4.0	108.2	7.0	107.6	6.8	106.1	6.7	4.4	7.8
sulfameter	106.7	11.6	101.1	17.4	99.9	8.3	0.8	3.1	100.9	6.5	105.2	4.3	101.0	4.6	2.9	4.8
sulfamethizole	75.5	4.3	113.3	3.3	93.5	2.1	3.7	5.3	81.6	3.6	93.8	3.7	103.8	4.5	1.9	3.3
sulfamethoxazole	116.0	9.6	104.4	8.8	108.4	3.0	2.0	5.0	101.0	6.2	113.2	6.3	96.5	4.6	2.3	9.1
sulfamethoxypyridazine	101.6	8.5	117.3	8.3	107.1	7.1	0.2	5.0	107.4	7.7	109.2	6.0	92.6	9.2	1.4	5.5
sulfamonnomethoxine	112.3	9.3	108.3	6.8	100.0	5.5	5.2	7.2	104.1	5.9	96.5	4.8	91.5	3.6	3.6	5.6
sulfamoxole	74.9	9.3	95.7	11.1	82.9	5.6	0.4	6.8	88.7	4.9	75.9	3.0	98.0	5.9	5.3	7.1
sulfaphenazole	96.5	7.8	117.8	6.0	108.6	2.2	0.8	1.0	106.0	6.6	101.7	4.9	102.2	4.1	2.7	4.9
sulfapyrazole	101.7	3.1	76.2	6.3	104.6	2.3	4.4	1.7	107.5	6.0	96.4	4.8	100.6	4.8	2.1	5.4
sulfapyridine	98.9	7.6	105.6	9.3	94.0	3.1	3.1	4.3	95.5	7.8	107.1	4.6	94.2	4.3	5.7	7.5
sulfaquinoxaline	72.6	10.7	109.1	7.1	88.8	5.2	1.2	3.0	89.5	6.1	74.2	6.1	96.8	3.1	3.7	8.7
sulfathiazole	77.3	3.7	115.3	6.0	86.1	3.7	0.1	2.1	112.0	3.0	97.8	4.2	97.7	5.1	4.7	8.7
sulfisomidine	76.0	6.3	114.1	6.2	87.3	2.8	1.6	1.9	90.6	6.7	96.1	6.2	114.0	5.1	3.7	14.6
sulfisoxazole	93.0	8.6	74.5	7.3	89.5	3.6	1.6	4.5	98.7	7.6	103.5	4.6	101.2	3.7	1.8	3.1
sulfadimethoxine	115.5	2.4	107.8	3.9	102.0	1.4	0.2	2.7	95.7	9.4	107.4	4.9	91.9	3.5	4.0	7.8
trimethoprim	100.3	3.4	99.7	3.8	98.5	3.8	0.6	2.2	85.5	6.0	94	4.0	90	4.2	3.1	4.5
